# How does cervical sagittal profile change after the spontaneous compensation of global sagittal imbalance following one- or two-level lumbar fusion

**DOI:** 10.1186/s12891-024-07518-7

**Published:** 2024-05-18

**Authors:** Chengxin Liu, Weiguo Zhu, Yongjin Li, Xiangyu Li, Bin Shi, Chao Kong, Shibao Lu

**Affiliations:** 1 Department of Orthopedics, Xuanwu Hospital, Capital Medical University, Beijing, China; 2 National Clinical Research Center for Geriatric Diseases, Beijing, China

**Keywords:** Cervical sagittal alignment, Sagittal imbalance, Lumbar fusion, Radiographic parameters

## Abstract

**Purpose:**

This study aimed to evaluate the cervical sagittal profile after the spontaneous compensation of global sagittal imbalance and analyze the associations between the changes in cervical sagittal alignment and spinopelvic parameters.

**Methods:**

In this retrospective radiographic study, we analyzed 90 patients with degenerative lumbar stenosis (DLS) and sagittal imbalance who underwent short lumbar fusion (imbalance group). We used 60 patients with DLS and sagittal balance as the control group (balance group). Patients in the imbalance group were also divided into two groups according to the preoperative PI: low PI group (≤ 50°), high PI group (PI > 50°). We measured the spinal sagittal alignment parameters on the long-cassette standing lateral radiographs of the whole spine. We compared the changes of spinal sagittal parameters between pre-operation and post-operation. We observed the relationships between the changes in cervical profile and spinopelvic parameters.

**Results:**

Sagittal vertical axis (SVA) occurred spontaneous compensation (*p* = 0.000) and significant changes were observed in cervical lordosis (CL) (*p* = 0.000) and cervical sagittal vertical axis (cSVA) (*p* = 0.023) after surgery in the imbalance group. However, there were no significant differences in the radiographic parameters from pre-operation to post-operation in the balance group. The variations in CL were correlated with the variations in SVA (*R* = 0.307, *p* = 0.041). The variations in cSVA were correlated with the variations in SVA (*R*=-0.470, *p* = 0.001).

**Conclusion:**

Cervical sagittal profile would have compensatory changes after short lumbar fusion. The spontaneous decrease in CL would occur in patients with DLS after the spontaneous compensation of global sagittal imbalance following one- or two-level lumbar fusion. The changes of cervical sagittal profile were related to the extent of the spontaneous compensation of SVA.

**Supplementary Information:**

The online version contains supplementary material available at 10.1186/s12891-024-07518-7.

No benefits in any form have been or will be received from a commercial party related directly or indirectly to the subject of this manuscript.

## Introduction

Sagittal imbalance is a general term encompassing spinal deformities with a significant manifestation of forward postural instability in standing, which conceptually results from a relative loss of lumbar lordosis, an increase in thoracic kyphosis, or combination of the two. Previous studies have shown that increasing sagittal vertical axis (SVA) was associated with poor preoperative and postoperative health-related quality-of-life (HRQOL) outcome scores [1, 2]. As a poorly tolerated and debilitating form of spinal deformity, sagittal imbalance is becoming an increasingly recognized cause of pain and disability in adults [1, 3]. When sagittal imbalance appears, several mechanisms from the upper part of the trunk to the lower limbs will be started to compensate this imbalance [4]. In addition, compensatory changes in cervical spine will be launched to maintain the horizontal sight.

Despite secondary to various spinal disorders, sagittal imbalance is common in elderly population due to degenerative changes of spine and the limited compensatory mechanism for degenerative lumbar stenosis (DLS) [5]. The sagittal imbalance in DLS might be a temporary lenitive or relieving posture for back pain. After lumbar decompression using short lumbar fusion, sagittal imbalance will appear spontaneous compensation [6]. Many studies described the compensatory changes that occurred in the cervical spine after thoracolumbar arthrodesis [7–14]. Nevertheless, there is a lack of study analyzing the variations of cervical sagittal alignment after the spontaneous compensation of sagittal imbalance following short lumbar fusion.

Therefore, we conduct this radiographic analysis in patients with DLS and sagittal imbalance who underwent one- or two-level lumbar fusion, with the aim to evaluate the cervical sagittal variations after the spontaneous compensation of global sagittal imbalance and analyze the associations between the changes in cervical sagittal alignment and spinopelvic parameters. In our study, we hypothesized that patients with DLS and positive sagittal imbalance partially compensate with increased cervical lordosis to maintain the horizontal gaze. In addition, we hypothesized that the spontaneous compensation of global sagittal imbalance following short lumbar fusion would generate a compensatory change in cervical alignment that results in a relaxation of the compensatory cervical hyperlordosis.

## Materials and methods

### Subjects

After approved by the institutional review board of our hospital, a retrospective radiographic analysis in patients with DLS and sagittal imbalance who underwent one- or two-level lumbar decompression and fusion at our center form January 2019 to December 2021 was performed. Inclusion criteria were: (1) aged > 18 years; (2) with spontaneous compensation of global sagittal imbalance after surgery (preoperative SVA > 50 mm, postoperative SVA ≤ 50 mm); (3) with complete radiographic imagings; (4) with a minimum follow-up of 3-month. Patients with ankylosing spondylitis, spinal tumor, spinal infection, or a history of spinal or pelvic surgery were excluded. Basic demographic data including age, gender, and body mass index (BMI) and surgical information were recorded.

### Radiographic measurements

**Fig. 1:**
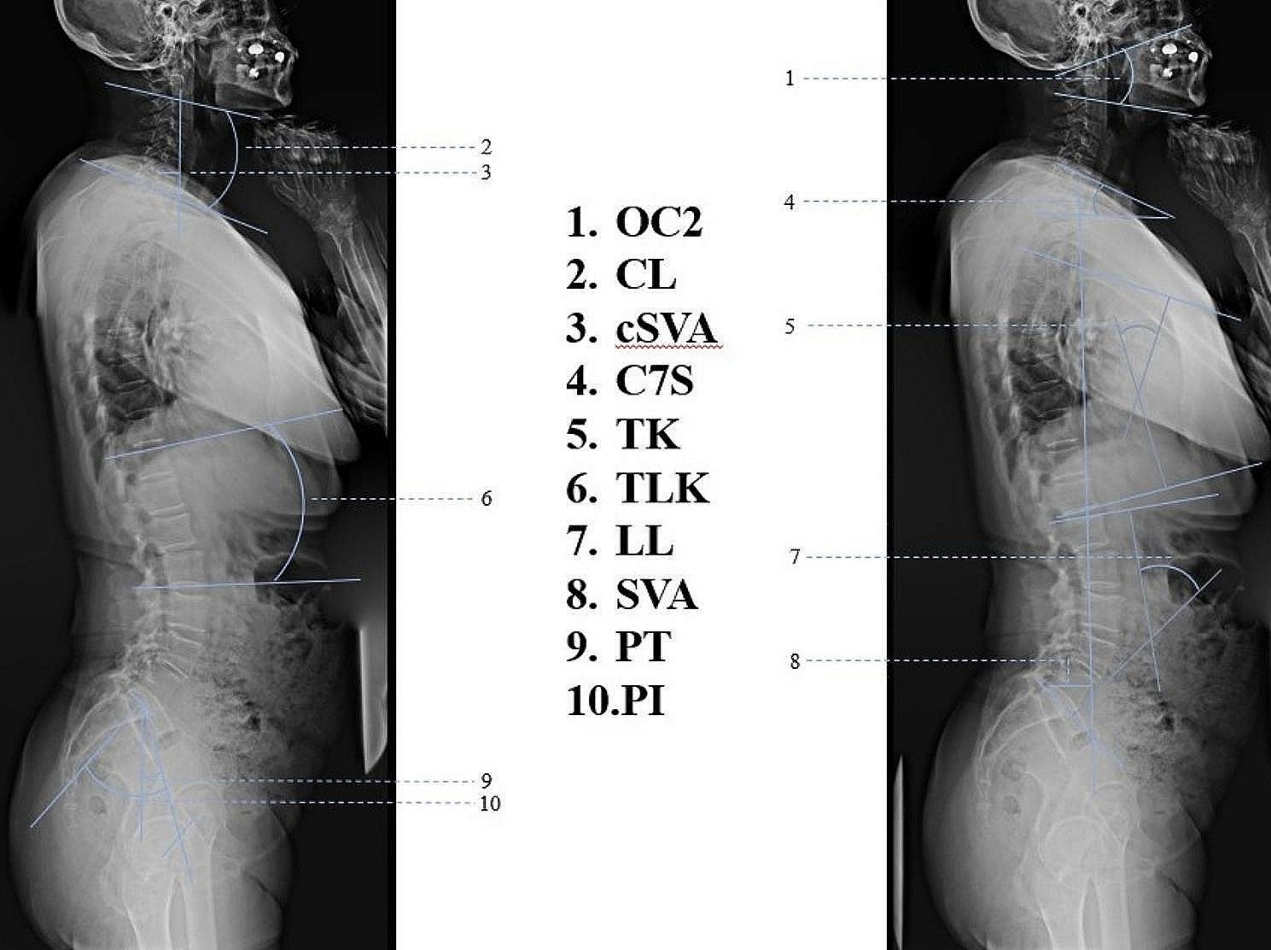
The measurement of sagittal parameters

The following radiographic parameters were measured on the long-cassette standing lateral radiographs of the whole spine before surgery and at the latest follow-up (Fig. 1): O-C2 angle (OC2, the angle between the McGregor line and the line drawn below C2 lower endplate), cervical lordosis (CL, was measured between the C2 lower endplate and the C7 lower endplate), cervical sagittal vertical axis (cSVA: the horizontal offset from a plumb line dropped from C2 vertebral body to the posterosuperior corner of C7 vertebra), C7 slope (C7S, the angle between a horizontal plane and a line parallel to the superior C7 endplate), thoracic kyphosis (TK, the angle between the upper endplate of T4 to the lower endplate of T12), thoracolumbar kyphosis (TLK, the angle between the upper endplate of T10 and the lower endplate of L2), lumbar lordosis (LL, the angle between the upper endplate of L1 and the upper endplate of S1 vertebra), pelvic incidence (PI, the angle between a line drawn from the center of the femoral head axis to the midpoint of the sacral plate and perpendicular to the sacral plate), pelvic tilt (PT, the angle between the lines connecting the midpoint of the sacral plate to the center of the femoral head axis and the vertical plane), sagittal vertical axis (SVA: the horizontal offset from a plumb line dropped from C7 vertebral body to the posterosuperior corner of S1 vertebra). We defined lordosis as a positive value and kyphosis as a negative value. We defined positive sagittal imbalance as SVA > 50 mm [13].

### Grouping

**Fig. 2:**
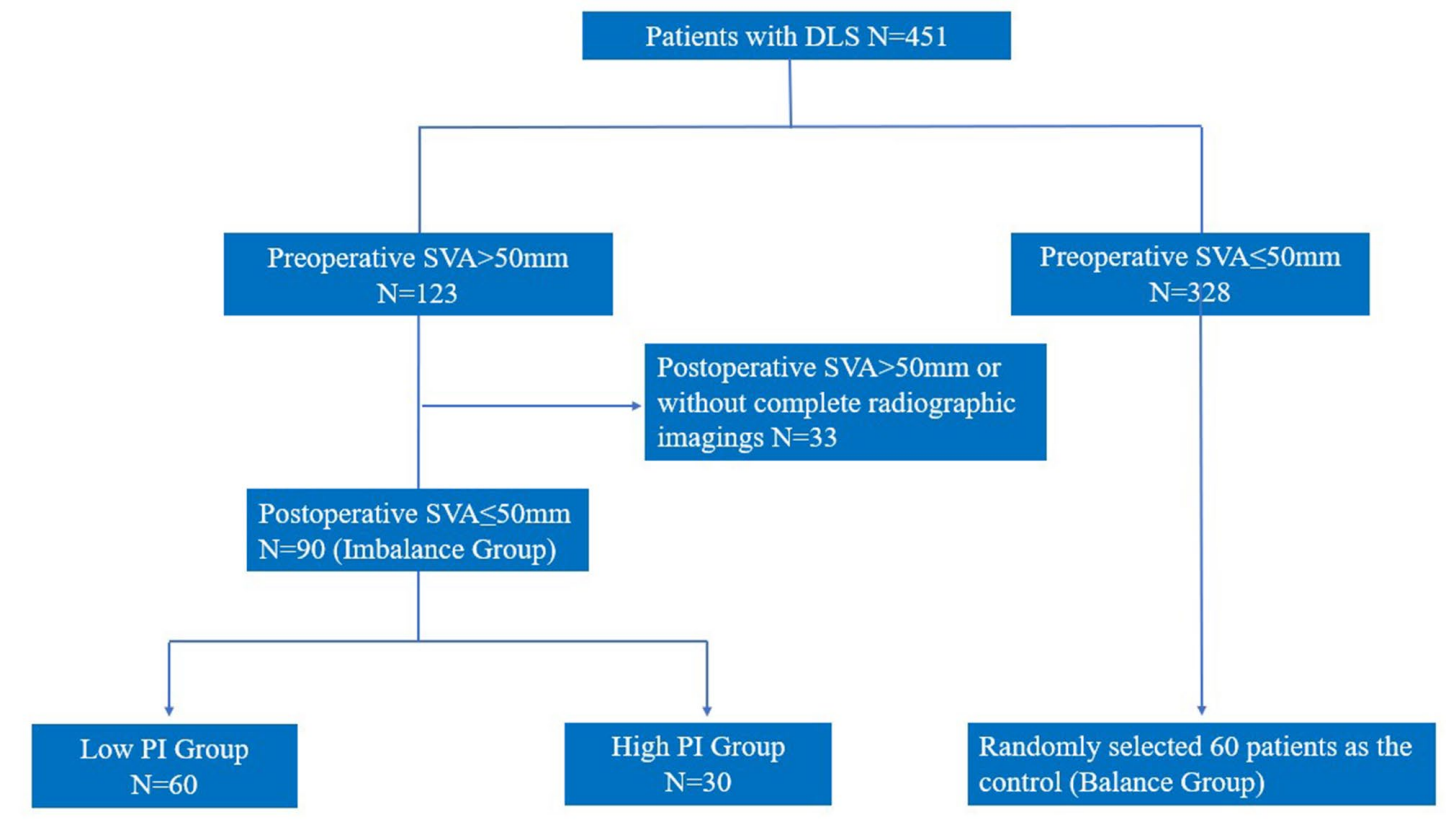
The flowchart of grouping. SVA: sagittal vertical axis; PI: pelvic incidence; DLS: degenerative lumbar stenosis

Variations in radiographic parameters (Δ) were calculated by subtracting the preoperative values from the values at the latest follow-up. Patients were divided into two groups according to the preoperative PI: low PI group (≤ 50°), high PI group (PI > 50°) [15]. In addition, sixty patients with DLS and normal sagittal profile (preoperative and postoperative SVA ≤ 50 mm) who underwent one- or two-level lumbar fusion were randomly (adopting the way of random numbers) selected to be included in the control group (balance group). The flowchart of patients grouping was showed in Fig. 2.

### Statistical analysis

All the data were presented as mean ± standard deviation and analyzed using SPSS version 22.0 software (SPSS, Inc, Chicago, IL). All sagittal parameters were measured twice and the mean value was used for final analysis. T-tests, Wilcoxon test and Chi-square test were used to assessed the differences of radiographic parameters and demographic data between the two groups. Pearson correlation analysis was used to analyze the correlations. P value < 0.05/n was considered as evidence of statistical significance and P value < 0.05 was considered as evidence of statistical suggestion.

## Results

### General information

A total of ninety DLS patients were included in the imbalance group (sixty-eight females and twenty-two males), with a mean age of 68.33±11.23 years, a mean BMI of 25.24±3.78 and a mean follow-up time of 127.62±25.08 days. Thirty-four patients received one-level lumbar fusion (37.78%) and fifty-six patients received two-level lumbar fusion (62.22%) (Table 1). A total of sixty patients were randomly selected as the control group (forty-four females and sixteen males), with a mean age of 64.50±8.79 years, a mean BMI of 24.85±3.18 and a mean follow-up time of 125.83±28.26 days. Twenty-four patients received one-level lumbar fusion (40%) and thirty-six patients received two-level lumbar fusion (60%) (Table 1). There were no significant differences in the age, sex, BMI, follow up time, preoperative PI, and surgical levels between the two groups (*p* > 0.05).

**Table 1:** Surgical levels

Parameters	Balance (*N* = 60)	Imbalance (*N* = 90)	*P* value
One level	24	34	*p* > 0.05
L5-S1	10	13	
L4-L5	11	15	
L3-L4	3	5	
L2-L3	0	1	
Two levels	36	56	*p* > 0.05
L4-S1	24	34	
L3-L5	12	20	
L2-L4	0	2	

*p* < 0.05 statistically significant difference

### Evaluation of preoperative and postoperative radiographic measurements

**Fig. 3:**
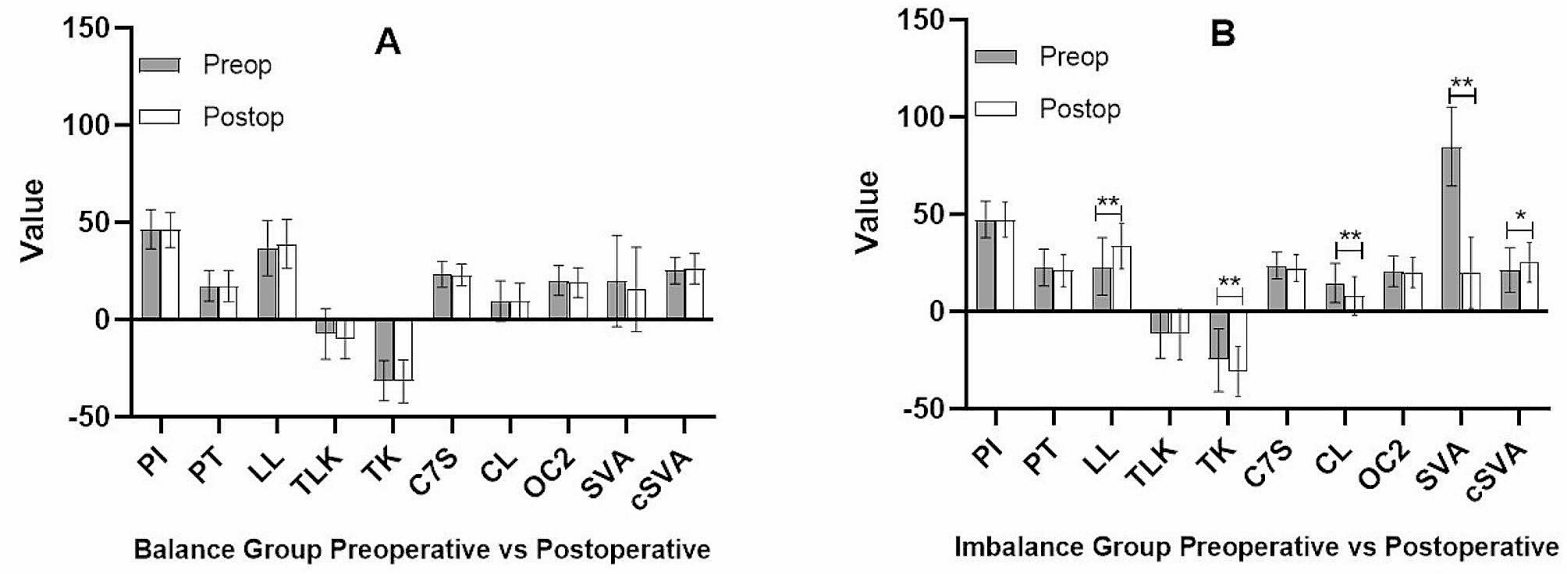
Comparison of preoperative and postoperative sagittal radiographic parameters in the balance and imbalance groups. Lordosis was defined as a positive value and kyphosis was defined as a negative value. PI: pelvic incidence, PT: pelvic tilt, LL: lumbar lordosis, TLK: thoracolumbar kyphosis, TK: thoracic kyphosis, C7S: C7 slope, CL: cervical lordosis, OC2: O-C2 angle, SVA: sagittal vertical axis, cSVA: cervical sagittal vertical axis. *p < 0.05, **p < 0.01statistically significant difference

In the balance group, there were no significant differences in the radiographic parameters from pre-operation to post-operation (Fig. 3-A). In the imbalance group, SVA was 84.91±20.32 mm before surgery and was spontaneous compensation to 19.97±18.15 mm after surgery (*p* = 0.000, Fig. 3-B). Besides, significant changes were also observed in LL, TK, CL and cSVA (Fig. 3-B). Especially, CL decreased from 14.67±10.04° to 8.03±9.90 ° (*p* = 0.000, ΔCL = 6.64±7.62°), cSVA increased from 21.35±11.43 mm to 25.31±10.30 mm (*p* = 0.023, ΔcSVA=-3.96±11.12 mm). Compared with the balance group, the postoperative variations in CL, TK, LL, and SVA were significantly higher in the imbalance group (Table 2).

**Table 2:** Variations in radiographic parameters

Parameters	Balance (*N* = 60)	Imbalance (*N* = 90)	*P* value
ΔOC2 (°)	1.14±5.81	0.55±6.07	*p* > 0.05
ΔCL (°)	-0.287±6.96	6.64±7.62	*P* = 0.001
ΔC7S (°)	0.35±5.70	1.23±5.05	*p* > 0.05
ΔcSVA (mm)	-1.01±6.73	-3.96±11.12	*p* > 0.05
ΔTLK (°)	2.49±8.79	0.15±3.59	*p* > 0.05
ΔTK (°)	0.33±6.99	5.72±12.43	*P* = 0.023
ΔLL (°)	-2.14±6.19	-10.66±9.11	*P* = 0.000
ΔPT (°)	0.18±4.09	1.56±5.56	*p* > 0.05
ΔSVA (mm)	4.31±23.32	64.95±28.54	*P* = 0.000

*p* < 0.05 statistically significant difference

PI: pelvic incidence, PT: pelvic tilt, LL: lumbar lordosis, TLK: thoracolumbar kyphosis, TK: thoracic kyphosis, C7S: C7 slope, CL: cervical lordosis, OC2: O-C2 angle, SVA: sagittal vertical axis, cSVA: cervical sagittal vertical axis

### Correlation analysis of patients in the imbalance group

The variations in CL (ΔCL) was suggestively correlated with PI (*R*=-0.300, *p* = 0.045) and ΔSVA (*R* = 0.307, *p* = 0.041) (Table 3). There were no significant correlations between the variations in OC2 (ΔOC2) and other radiographic parameters (Table 4). The variations in cSVA (ΔcSVA) was significantly correlated with ΔSVA (*R*=-0.470, *p* = 0.001), ΔLL (*R* = 0.493, *p* = 0.001) and ΔTK (*R*=-0.439, *p* = 0.004) (Table 5).

**Table 3:** Correlations between radiographic parameters and the variations in CL for 90 patients with spontaneous compensation of global sagittal imbalance after surgery

Parameters	Pearson	*P* value
Age (years)	-0.185	0.224
BMI	-0.147	0.336
Follow-up (days)	0.099	0.517
PI (°)	-0.300	0.045*
ΔSVA (mm)	0.307	0.041*
ΔPT (°)	0.090	0.557
ΔLL (°)	-0.223	0.181
ΔTLK (°)	0.073	0.632
ΔTK (°)	0.123	0.422

**p < 0.0055 for statistically significance and *p < 0.05 for statistically suggestion

PI: pelvic incidence, PT: pelvic tilt, LL: lumbar lordosis, TLK: thoracolumbar kyphosis, TK: thoracic kyphosis, cSVA: cervical sagittal vertical axis, SVA: sagittal vertical axis. Δ: variations in radiographic parameters

**Table 4:** Correlations between radiographic parameters and the variations in OC2 for 90 patients with spontaneous compensation of global sagittal imbalance after surgery

Parameters	Pearson	*P* value
Age (years)	0.190	0.212
BMI	-0.016	0.915
Follow-up (days)	-0.054	0.724
PI (°)	-0.228	0.132
ΔSVA (mm)	0.010	0.947
ΔPT (°)	-0.128	0.403
ΔLL (°)	-0.051	0.738
ΔTLK (°)	0.090	0.556
ΔTK (°)	-0.126	0.409

**p < 0.0055 for statistically significance and *p < 0.05 for statistically suggestion

PI: pelvic incidence, PT: pelvic tilt, LL: lumbar lordosis, TLK: thoracolumbar kyphosis, TK: thoracic kyphosis, cSVA: cervical sagittal vertical axis, SVA: sagittal vertical axis. Δ: variations in radiographic parameters

**Table 5:** Correlations between radiographic parameters and the variations in cSVA for 90 patients with spontaneous compensation of global sagittal imbalance after surgery

Parameters	Pearson	*P* value
Age (years)	0.233	0.124
BMI	0.003	0.986
Follow-up (days)	-0.059	0.702
PI (°)	0.073	0.633
ΔSVA (mm)	-0.470	0.001**
ΔPT (°)	-0.145	0.343
ΔLL (°)	0.493	0.001**
ΔTLK (°)	-0.178	0.243
ΔTK (°)	-0.439	0.004**

**p < 0.0055 for statistically significance and *p < 0.05 for statistically suggestion

PI: pelvic incidence, PT: pelvic tilt, LL: lumbar lordosis, TLK: thoracolumbar kyphosis, TK: thoracic kyphosis, cSVA: cervical sagittal vertical axis, SVA: sagittal vertical axis. Δ: variations in radiographic parameters

### Comparison of patients in the imbalance group according to the value of pelvic incidence

**Fig. 4:**
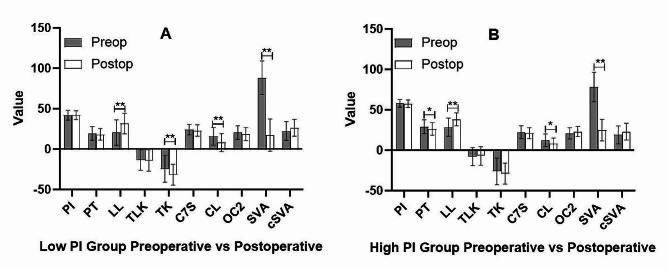
Comparison of preoperative and postoperative sagittal radiographic parameters in the low PI and high PI groups. Lordosis was defined as a positive value and kyphosis was defined as a negative value. PI: pelvic incidence, PT: pelvic tilt, LL: lumbar lordosis, TLK: thoracolumbar kyphosis, TK: thoracic kyphosis, C7S: C7 slope, CL: cervical lordosis, OC2: O-C2 angle, SVA: sagittal vertical axis, cSVA: cervical sagittal vertical axis. *p < 0.05, **p < 0.01statistically significant difference

A total of sixty DLS patients were included in the low PI group (forty-two females and eighteen males), with a mean age of 68.67±13.04 years, a mean BMI of 25.09±3.57 and a mean follow-up time of 129.93±27.41 days. Twenty-four patients received one-level lumbar fusion (40%) and thirty-six patients received two-level lumbar fusion (60%). A total of thirty patients were included in the high PI group (twenty-six females and four males), with a mean age of 67.67±6.54 years, a mean BMI of 25.54±3.92 and a mean follow-up time of 123.00±19.65 days. Ten patients received one-level lumbar fusion (33.33%) and twenty patients received two-level lumbar fusion (66.67%). There were no significant differences in the age, sex, BMI, follow up time, and surgical levels between low PI and high PI groups. In the low PI group, SVA was spontaneous compensation after surgery (pre 88.26±20.76 mm VS post 17.45±19.79 mm, *p* = 0.000). LL increased from 20.43±15.64° to 31.55±12.63 ° (*p* = 0.000). TK increased from 24.57±16.27° to 31.72±12.91 ° (*p* = 0.009, kyphosis was defined as a negative value). CL decreased from 15.93±10.83° to 8.12±11.08° (*p* = 0.000). There were no significant changes in PI, PT, TLK, C7S, OC2, and cSVA between pre-operation and post-operation (Fig. 4-A). In the high PI group, SVA was also spontaneous compensation after surgery (pre 78.23±18.25 mm VS post 24.86±13.24 mm, *p* = 0.000). PT decreased from 29.08.±8.48° to 26.44±7.85 ° (*p* = 0.037). LL increased from 28.52.13±11.32° to 38.27±7.91 ° (*p* = 0.000). CL decreased from 12.14±7.96° to 7.85±7.30 ° (*p* = 0.020). There were no significant changes in PI, TLK, TK C7S, OC2 and cSVA between pre-operation and post-operation (Fig. 4-B).

## Discussion

Alteration of normal cervical sagittal alignment are related to neck pain, disc degeneration, radiculopathy, myelopathy, and outcomes of cervical surgery [16–22]. Plenty of studies reported that CL would decrease when sagittal deformity and imbalance were corrected with long fusion [9–11, 13]. The alteration of CL after long fusion was correlated with the location upper instrumented vertebra [11], and the extent of sagittal deformity correction [13]. In the study by Nasto et al. [8], there was a significant decrease of CL in thoracic deformity patients and a significant increase of CL was observed in thoracolumbar deformity patients after a posterior-only correction surgery. Ha et al. [14] grouped patients by low SVA (preoperative SVA ≤ 6 cm) and high SVA (preoperative SVA ≥ 9 cm). In the low SVA group, CL significantly increased after corrective thoracolumbar deformity surgery, while in the high SVA group, a decrease CL was observed. These findings suggested that the magnitude of CL was associated with global changes.

 [23] In order to relieve neural compression, patients with DLS tend to adapt a forward bending posture which may induce sagittal imbalance. Buckland et al. [24] reported that patients with DLS permit anterior truncal inclination and recruit posterior pelvic shift instead of PT to maintain sagittal balance. For patients with DLS, the forward bending posture and sagittal imbalance will be spontaneous compensation after decompression following short lumbar fusion. Our study reported the consistent results that SVA decreased significantly after surgery [6]. In our study, we found significant changes in LL, TK, CL and cSVA after spontaneous compensation of sagittal imbalance in the imbalance group, while no significant changes in sagittal alignment were detected from pre-operation to post-operation in the balance group (Fig. 3). In order to adapt to the spontaneous compensation in sagittal imbalance, postoperative TK and cSVA significantly increased, and postoperative CL significantly decreased (Table 2). In the present study, we also observed that preoperative CL was larger in the imbalance group than in the balanced group (14.67±10.04° VS 9.49±10.47°, *p* = 0.035). Meanwhile, CL decreased in patients with sagittal imbalance and there was no significant difference in CL (8.03±9.90° VS 9.78±8.93°, *p* = 0.438) between the two groups after surgery. These data suggested that cervical hyper-lordosis would disappear and cervical curvature would recovered the normal after the spontaneous compensation of global sagittal imbalance following short lumbar fusion in the patients with DLS.

In Ha et al. study [14], T1 slope (T1S) was described as a driving force for inducing reciprocal alterations in cervical spine. Other studies also reported the changes of T1S after correcting spinal imbalance [7, 8, 13]. However, our study didn’t show the changes of C7S after spontaneous compensation sagittal imbalance (C7S can substitute T1S on radiographic images [25, 26]). This is likely attributable to the fact that the reciprocal changes can be distributed throughout the unfused thoracic and cervical spine. Long fusion terminates in the thoracic spine and even upper thoracic spine. Conversely, our study mainly included low lumbar fusion. Low lumbar fusion leaves large room for thoracolumbar and thoracic adaptation, whereas a long fusion does not. Therefore, C7S may not show significant changes after surgery even if sagittal imbalance is spontaneously compensation after short lumbar fusion. The present study, in combination with prior studies [8, 12, 14], indicated that cervical compensatory mechanisms are not all the same in the patients with different sagittal imbalance.

Neuman et al. [11] and Smith et al. [13] emphasized the important role of ΔSVA in the variations in cervical alignment after correcting spinal imbalance. Our study found that the variations in CL (ΔCL, *r* = 0.307, *p* = 0.041) and cSVA (ΔcSVA, *r*=-0.470, *p* = 0.001) were correlated with ΔSVA. The present study, in combination with prior studies, showed that the more changes of postoperative SVA, the greater variations may appear in the cervical alignment after surgery.

Our study reported the correlation between preoperative PI and ΔCL (*r*=-0.300, *p* = 0.045). Considering the important role of PI in pelvic compensation, patients in the imbalance groups were divided into two groups according to preoperative PI (Fig. 4). We found that postoperative change in cervical alignment was not associated with PI and a significant decrease of CL would occur in the patients with low or high PI after spontaneous compensation of global sagittal imbalance (a significant decrease of CL was observed in two groups). However, different postoperative changes were discovered in PT and TK. A significant increase of TK (ΔTK = 7.15±14.05°) was observed in low PI group and a significant decrease of PT (ΔPT = 2.64±4.45°) was observed in high PI group after surgery. These findings demonstrated that different compensatory mechanisms appear in spinopelvic alignment when sagittal imbalance occurs in patients with DLS and different PI. In addition to anterior truncal inclination and posterior pelvic shift, pelvic retroversion is more common in patients with large PI and thoracic extension is more common in patients with low PI. However, cervical hyper-lordosis will appear in both groups when sagittal imbalance occurs in patients with DLS. For patients with DLS, cervical alignment was affected by the sagittal balance of the whole spine rather than thoracic or spinopelvic regional alignment. The spontaneous decrease in CL would occur after the spontaneous compensation of global sagittal imbalance following one- or two-level lumbar fusion.

**Fig. 5:**
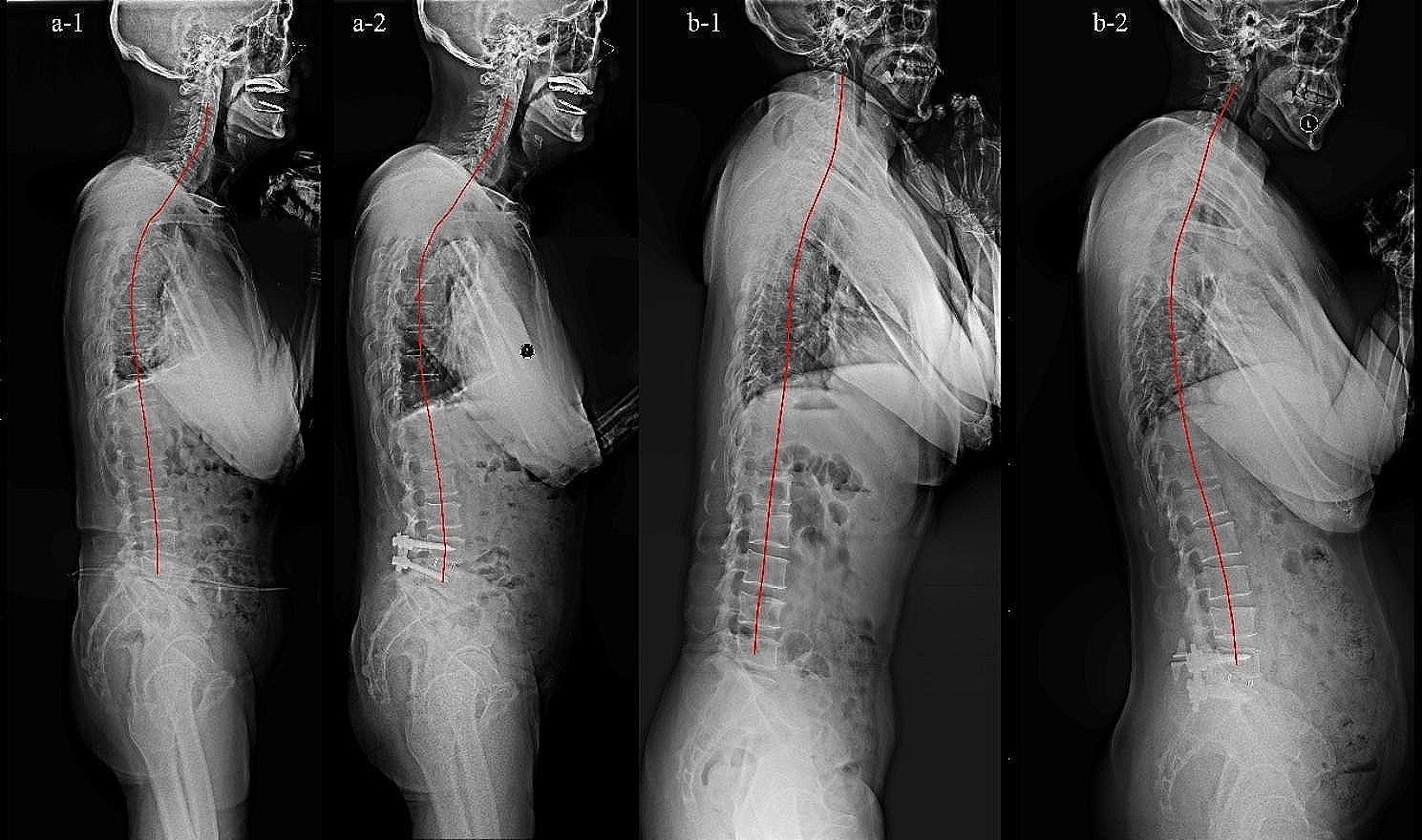
Case illustrations. Cervical sagittal alignment didn’t appear spontaneous variation after surgery. (Fig. 5-a1, a2): **a-1** Sagittal preoperative radiographs; **a-2** Sagittal postoperative radiographs of the same patient after one level posterior lumbar fusion (L4-L5). Cervical sagittal alignment appeared spontaneous variation following spontaneous compensation of sagittal imbalance after surgery (Fig. 5-b1, b2): **b-1** Sagittal preoperative radiographs; **b-2** Sagittal postoperative radiographs of the same patient after one level posterior lumbar interbody fusion (L5-S1).

To our best knowledge, the present study is the first study to describe the compensatory changes of cervical sagittal alignment in patients with DLS after the spontaneous compensation of global sagittal imbalance following short-segment lumbar fusion. Our present study provided evidences that CL would decrease significantly and cSVA would increase significantly after the spontaneous compensation of sagittal imbalance (Fig. 5). This will improve the global understanding of sagittal interactions as an aid to patient evaluation, surgical planning, and prevention of secondary cervical spine disorders. There are also some limitations in our study. First, we didn’t get clinical score for cervical spine and could not compare the variations of cervical clinical symptoms between pre-operation and post-operation. Second, the patients in this study were main older people. Therefore, some patients maybe had cervical spine degeneration in different degrees. Third, the study was a small sample exploratory study, the follow-up time and the sample size were relatively insufficient.

## Conclusion

This study first demonstrated that cervical sagittal profile would have compensatory changes after short lumbar fusion. The spontaneous decrease in CL would occur in patients with DLS after the spontaneous compensation of global sagittal imbalance following one- or two-level lumbar fusion. The changes of cervical sagittal profile were related to the extent of the spontaneous compensation of SVA.

### Electronic supplementary material

Below is the link to the electronic supplementary material.

Supplementary Material 1

Supplementary Material 2

Supplementary Material 3

## Data Availability

The datasets generated and/or analysed during the current study are not publicly available due [This study is part of a series of studies that have not been completely completed] but are available from the corresponding author on reasonable request.

## Abbreviations

DLS: Degenerative lumbar stenosis
HRQOL: Health-related quality-of-life
BMI: Body mass index
OC2: O-C2 angle
CL: Cervical lordosis
cSVA: Cervical sagittal vertical axis
C7S: C7 slope
T1S: T1 slope
TK: Thoracic kyphosis
TLK: Thoracolumbar kyphosis
LL: Lumbar lordosis
PI: Pelvic incidence
PT: Pelvic tilt
SVA: Sagittal vertical axis
