# Supplementary material for: How does cervical sagittal profile change after the spontaneous compensation of global sagittal imbalance following one- or two-level lumbar fusion
Source: BMC Musculoskelet Disord. 2024 May 18;25:387. doi: 10.1186/s12891-024-07518-7 (PMC11102194; doi:10.1186/s12891-024-07518-7)
Supplement: Supplementary file 1 — Supplementary Material 1 [file 12891_2024_7518_MOESM1_ESM.doc]

**Table 7**. Comparison of cervical parameters between low PI and high PI groups

| **Parameters** | **Low PI group (N=60)** | **High PI group (N=30)** | **P** |
| --- | --- | --- | --- |
| **Pre CL (°)** | 15.93±10.83 | 12.14±7.96 | 0.236 |
| **Post CL (°)** | 8.12±11.08 | 7.85±7.30 | 0.933 |
| **Pre cSVA (mm)** | 22.49±11.57 | 19.08±11.21 | 0.351 |
| **Post cSVA (mm)** | 26.44±10.28 | 23.06±10.30 | 0.305 |
| **Pre OC2 (°)** | 20.53±8.44 | 20.85±6.88 | 0.901 |
| **Post OC2 (°)** | 18.55±8.10 | 23.15±6.31 | 0.061 |

p<0.05 statistically significant difference

CL: cervical lordosis, OC2: O-C2 angle, cSVA: cervical sagittal vertical axis
