# Supplementary material for: How does cervical sagittal profile change after the spontaneous compensation of global sagittal imbalance following one- or two-level lumbar fusion
Source: BMC Musculoskelet Disord. 2024 May 18;25:387. doi: 10.1186/s12891-024-07518-7 (PMC11102194; doi:10.1186/s12891-024-07518-7)
Supplement: Supplementary file 2 — Supplementary Material 2 [file 12891_2024_7518_MOESM2_ESM.doc]

**Table 6**. comparison of cervical parameters between balance and imbalance groups

| **Parameters** | **Balance group (N=60)** | **Imbalance group (N=90)** | **P** |
| --- | --- | --- | --- |
| **Pre CL (°)** | 9.49±10.47 | 14.67±10.04 | **0.035** |
| **Post CL (°)** | 9.78±8.93 | 8.03±9.90 | 0.438 |
| **Pre cSVA (mm)** | 25.20±6.79 | 21.35±11.43 | 0.072 |
| **Post cSVA (mm)** | 26.21±7.76 | 25.31±10.30 | 0.688 |
| **Pre OC2 (°)** | 20.19±7.69 | 20.64±7.88 | 0.809 |
| **Post OC2 (°)** | 19.05±7.67 | 14.66±10.04 | 0.576 |

p<0.05 statistically significant difference

CL: cervical lordosis, OC2: O-C2 angle, cSVA: cervical sagittal vertical axis
